# Role of blue and red light in stomatal dynamic behaviour

**DOI:** 10.1093/jxb/erz563

**Published:** 2019-12-24

**Authors:** Jack S A Matthews, Silvere Vialet-Chabrand, Tracy Lawson

**Affiliations:** 1 School of Life Sciences, University of Essex, Wivenhoe Park, Colchester, UK; 2 Research School of Biology, Australian National University, Australia

**Keywords:** Blue light, guard cells, mesophyll, osmoregulation, photosynthesis, red light, signalling, stomata

## Abstract

Plants experience changes in light intensity and quality due to variations in solar angle and shading from clouds and overlapping leaves. Stomatal opening to increasing irradiance is often an order of magnitude slower than photosynthetic responses, which can result in CO_2_ diffusional limitations on leaf photosynthesis, as well as unnecessary water loss when stomata continue to open after photosynthesis has reached saturation. Stomatal opening to light is driven by two distinct pathways; the ‘red’ or photosynthetic response that occurs at high fluence rates and saturates with photosynthesis, and is thought to be the main mechanism that coordinates stomatal behaviour with photosynthesis; and the guard cell-specific ‘blue’ light response that saturates at low fluence rates, and is often considered independent of photosynthesis, and important for early morning stomatal opening. Here we review the literature on these complicated signal transduction pathways and osmoregulatory processes in guard cells that are influenced by the light environment. We discuss the possibility of tuning the sensitivity and magnitude of stomatal response to blue light which potentially represents a novel target to develop ideotypes with the ‘ideal’ balance between carbon gain, evaporative cooling, and maintenance of hydraulic status that is crucial for maximizing crop performance and productivity.

## Teaser

We review stomatal signalling and osmoregulatory processes in response to red and blue light, with the aim of tuning stomatal sensitivity to enhance crop performance.

## Introduction

Stomata control the flux of CO_2_ into the leaf and water lost through transpiration, and are crucial in maintaining plant water status, leaf temperature, and photosynthetic rates, depending on the current needs of the plant. The surface of most leaves is effectively impermeable to water and CO_2_; therefore, most of the CO_2_ fixed and water lost by plants must pass through stomatal pores (Cowan and Troughton, 1971; Caird *et al.*, 2007; Jones, 2013), with stomata controlling the majority of gas exchange between the leaf and external environment, despite typically occupying only a small proportion (0.3–5%) of the leaf surface (Morison, 2003). The capacity of stomata to allow CO_2_ into or water out of the leaf is known as stomatal conductance (*g*_s_), with stomatal behaviour leading to alterations in stomatal aperture and therefore diffusional fluxes. Stomatal aperture is governed by changes in guard cell (GC) volume and turgor pressure driven by alterations in osmotic potential (Willmer and Fricker, 1996; Blatt, 2000; Chen *et al.*, 2012), and, along with stomatal density, determines *g*_s_. Adjustment in stomatal behaviour is driven by the external environmental (e.g. light) and internal signalling cues (see Lawson and Blatt, 2014), with responses to these signals varying between and within species (Aasamaa and Sõber, 2011; Drake *et al.*, 2013; McAusland *et al.*, 2016; Matthews *et al.*, 2018). In general, stomatal opening is triggered by increasing light or temperatures (up to an optimum), low CO_2_, and low vapour pressure deficit (VPD), whilst closure is driven by the reverse; decreasing light, extreme low or high temperatures, high CO_2_, and high VPD (Raschke, 1975; Outlaw, 2003; Ainsworth and Rogers, 2007; Bernacchi *et al.*, 2007; Matthews and Lawson, 2019). In response to changes in these environmental cues, various ion and solute channels in GCs are activated via a signalling cascade, triggering the uptake or release of ions and solutes that modify the osmotic and water potential of the cell, leading to the uptake or loss of water, changes in turgor pressure, and, therefore, changes in stomatal aperture (e.g. see Willmer and Fricker, 1996; Shimazaki, 2007; Lawson and Blatt, 2014; Inoue and Kinoshita, 2017). It is well established that high *g*_s_ can facilitate a higher net photosynthetic rate (*A*); however, this is at a greater cost of water loss, making plants more vulnerable to water stress or cavitation (depending on the species) (Naumburg and Ellsworth, 2000; Lawson *et al.*, 2010, 2012; Matthews *et al.*, 2017), whereas low *g*_s_ can limit CO_2_ diffusion and photosynthetic rates by up to 20% in well-watered C_3_ species, negatively affecting biomass accumulation and yield (Farquhar and Sharkey, 1982; Barradas *et al.*, 1994; Fischer *et al.*, 1998; Lawson *et al.*, 2010). Low stomatal aperture may also impact evaporative cooling and conservation of leaf temperature in an optimal range, which is important for maintaining photosynthetic rates and therefore has consequences for harvestable yield (Fischer *et al.*, 1998; Fischer and Rebetzke, 2018; Matthews and Lawson, 2019). Although it is well established that a close correlation between *g*_s_ and *A* exists, and whilst the exact signalling pathways and mechanisms that support this relationship have not been fully established, several theories have been put forward. This correlation between *A* and *g*_s_ is understood to exist to optimize the trade-off between carbon gain and water loss (Wong *et al.*, 1979; Mansfield *et al.*, 1990; Buckley and Mott, 2013; Buckley, 2017), with stomata continually adjusting aperture to balance the requirement for CO_2_ for photosynthesis against the need to maintain leaf hydration. However, stomatal responses tend to be an order of magnitude slower than photosynthetic responses, and this leads to non-coordinated responses in *g*_s_ and *A* (Lawson *et al.*, 2010; McAusland *et al.*, 2016), where lags in behaviour or slow stomatal responses will often lead to a limitation in carbon gain or unnecessary increase in water loss (see Lawson and Vialet-Chabrand, 2019). It has therefore been suggested that species with more rapid *g*_s_ responses to changing environmental conditions will maximize both photosynthesis and water use efficiency (WUE; Lawson *et al.*, 2010; Raven, 2014; Lawson and Blatt, 2014; Vialet-Chabrand *et al.*, 2017a; Lawson and Vialet-Chabrand, 2019) and that manipulation of stomatal kinetics could be a novel approach to enhancing CO_2_ uptake, maintaining optimal leaf temperature for carbon assimilation, and improving water use in important crops (Lawson *et al.*, 2010; Faralli *et al.*, 2019), particularly given the predicted changes to the climate (Matthews and Lawson, 2019).

As photosynthesis and stomata do not respond with the same rapidity to changes in light intensity and spectral quality (Shimazaki *et al.*, 2007), short-term fluctuations in light lead to temporal and spatial disconnections between stomatal behaviour and photosynthesis (e.g. Kirschbaum *et al.* 1988; Vialet-Chabrand *et al.*, 2017b; Lawson *et al.*, 2018; Matthews *et al.*, 2018). Although it is possible to detect these spatial and temporal differences in *g*_s_ and *A* (e.g. using imaging approaches; see McAusland *et al.*, 2013; Vialet-Chabrand and Lawson, 2019), there are still major gaps in our understanding of the impact of this variation on plant carbon gain or WUE and how such patterns relate to differences in light perception, signal transduction, and stomatal behaviour. Therefore, manipulation of stomatal behaviour and/or the mechanisms that coordinate stomatal response to light quality and intensity could provide potential targets for increasing photosynthesis, WUE, and overall plant productivity in the field. However, in order to succeed, more evidence on the mechanisms and signalling pathways associated with stomatal dynamics in response to light quantity and quality is required, as well as an understanding of the influence of the mesophyll and the hierarchy of stomatal responses.

### Diurnal changes in light quality and intensity impact stomatal behaviour

**Fig. 1.**
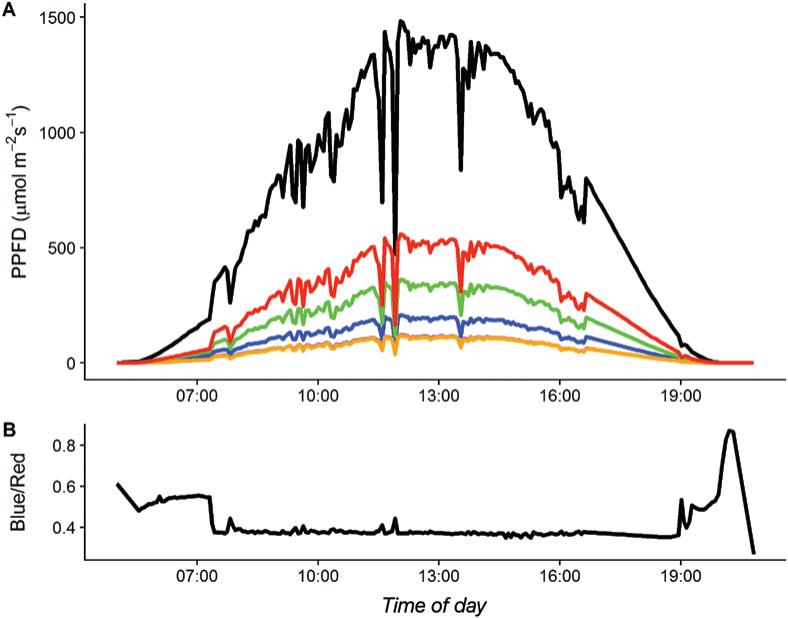
Diurnal variation in total irradiance (black) and spectral composition: 360–450 nm (purple), 450–500 nm (blue), 500–570 nm (green), 570–591 nm (yellow), 591–610 nm (orange), and 610–760 nm (red) (A); including changes in the ratio of blue:red light, highlighting peaks in blue light at the beginning and end of the day (B). Purple, yellow, and orange lines almost entirely overlap. Spectral measurements were performed using a microspectrometer C12880MA attached to a C13016 circuit and calibrated to provide PAR intensity

Plants experience light in a range of intensities and spectral properties, largely due to passing clouds, changes in canopy cover, and self-shading from overlapping leaves, and this produces unpredictable fluctuations in spectral distribution (see Fig. 1) that impact stomatal behaviour, carbon gain, and the diurnal course of WUE (Pearcy, 1990; Chazdon and Pearcy, 1991; Kaiser and Kappen, 2000, 2001; Vialet-Chabrand *et al.*, 2016; Matthews *et al.*, 2017, 2018). The path of the sun across the sky affects both the quantity and quality of the light available to plants at any given location. At dawn and towards dusk as solar angle diminishes, sunlight negotiates an increasingly long path through the atmosphere, enhancing atmospheric light absorption and scattering, thus depleting shorter wavelengths of light (Kendrick and Kronenberg, 1994). Furthermore, the contribution of direct radiation relative to diffuse radiation declines, often leading to a pronounced peak in blue light (Urban *et al.*, 2007), changing the light quality and therefore plant response. Changes in the quality of light throughout the day may impact stomatal dynamic response and diurnal behaviour, and therefore affect photosynthetic efficiency and water use through *g*_s_ enforced diffusional constraints on *A*.

## Stomatal response to light

Stomata in C_3_ and C_4_ species open in response to increasing light intensity whilst closure is brought about by reductions in intensity, whereas CAM (crassulacean acid metabolism) species display the opposite response, with stomata opening in darkness for nocturnal CO_2_ uptake and closing in the light (Cockburn, 1983). The stomatal opening responses to light can be divided into two distinct pathways; termed the red or mesophyll/photosynthetic response (herein, termed the red light response) and the GC-specific blue light response (Zeiger, 1983; Assmann and Shimazaki, 1999; Roelfsema and Hedrich, 2005; Shimazaki *et al.*, 2007; Doi *et al.*, 2015; Inoue and Kinoshita, 2017). The red light response occurs at high fluence rates and saturates at similar intensities to photosynthesis, and many studies have suggested that response is the primary mechanism linking stomatal behaviour with photosynthetic rates and that it is responsible for the close correlation between *A* and *g*_s_ (Wong *et al.*, 1979; Ball and Berry, 1982). The blue light stomatal response occurs and is saturated at a low fluence rate (~5–10 µmol m^–2^ s^–1^; Shimazaki *et al.*, 2007), is GC specific, and is thought to be independent of mesophyll photosynthesis. Blue-light-initiated responses are not exclusive to stomata, and other responses are initiated by this signal that are important for optimal performance; including phototropism (see Christie and Briggs, 2001; Briggs and Christie, 2002; Christie, 2007), photomorphogenesis (Lin and Shalitin, 2003), flowering and circadian clock function (Banerjee and Batschauer, 2005), and the directional movement of chloroplasts in the mesophyll and GC complexes (see Haupt, 1999; Banaś *et al.*, 2012).

Although most research on the spectral aspect of stomatal behaviour and photosynthesis focuses on red and blue light, there is also evidence that green light plays a vital role in physiological responses to the environment. It has been reported that plants may use green wavelengths as a crucial signal to determine short-term dynamic responses and long-term developmental acclimation, enabling optimization of resource use efficiency and photosynthesis to available irradiance (Smith *et al.*, 2017). Furthermore, green light has been shown to inhibit blue-light-induced stomatal opening (Talbott *et al.*, 2006; Aasamaa and Aphalo, 2016), potentially to prevent excessive leaf water loss in shade environments when photosynthetic potential is low (Talbott *et al.*, 2006). In this review, we focus on light-stimulated stomatal behaviour and specifically on the red-(photosynthetic) and blue-light-driven responses, what is understood about the different signalling pathways involved, and GC metabolism that facilitates these responses. We further explore how a better understanding of stomatal response to irradiance, and the influence of the mesophyll on these responses, could provide novel targets for the development of plants with improved photosynthetic carbon gain and WUE.

## Red light response of stomata

**Fig. 2.**
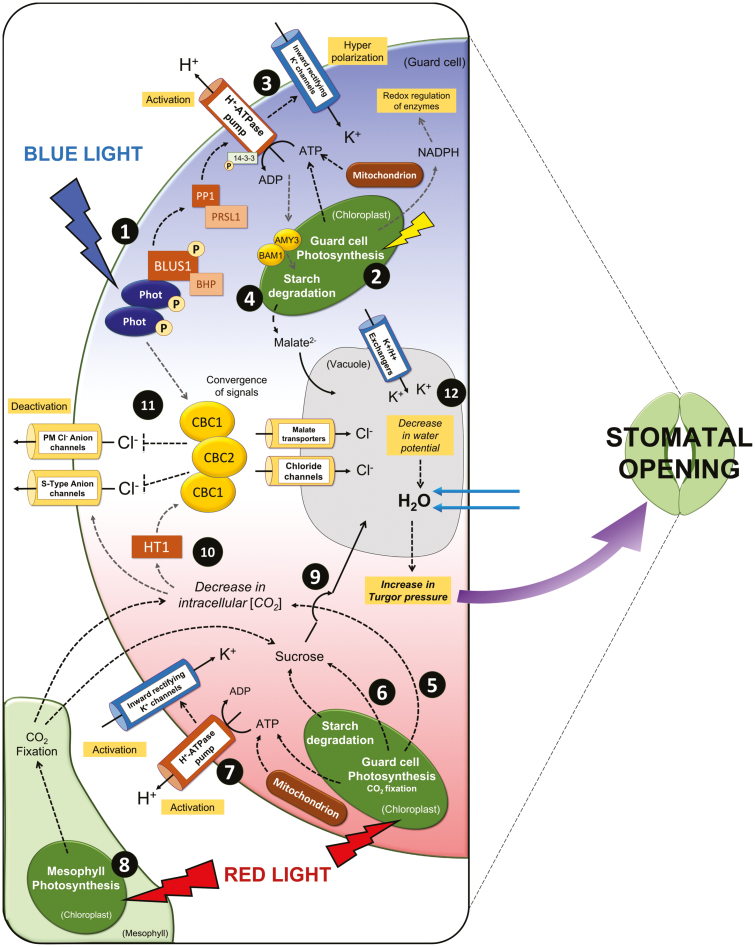
Signalling pathways for blue and red light in stomatal guard cells. Signalling steps involved in stomatal response to blue light (1–4, 11–12) and red light (5–10, 11–12). Phototropins are activated by blue light, phosphorylating BLUS1 and downstream kinases, leading to activation of H^+^-ATPase protein pumps (1). Guard cell (GC) photosynthesis in the chloroplast provides ATP for H^+^-ATPase pump activation; ATP is also provided by GC mitochondria (2). Activation of inward-rectifying K^+^ channels leads to accumulation of K^+^ in the GC (3). Production of the counter-ion malate^2+^ via starch degradation, glycolysis, and PEPc activation (4). Red-light-induced GC photosynthesis decreases the intracellular CO_2_ concentration (*C*_i_) (5), increases GC sucrose content (6), and induces plasma membrane H^+^-ATPase phosphorylation (7). Mesophyll photosynthesis further reduces *C*_i_ and provides sucrose that accumulates in the GC (8), which is then transported to the vacuole (9). A low *C*_i_ signal induced by red light deactivates Cl^–^ and S-type anion channels via HT1 protein kinase and CBC1/2 (10), linking blue light (via phototropins) with red-light-driven signalling networks, increasing Cl^–^ accumulation in the GC (11). Transport and accumulation of K^+^, the counter-ions Cl^–^ and malate^2–^, and sucrose into the vacuole occurs via K^+^/H^+^ exchangers and chloride channel malate transporters, decreasing GC water potential, and increasing water uptake and turgor pressure, leading to stomatal opening (12). Dashed arrows indicate the direction of the signal (may involve several steps). Full arrows indicate the direction of ion movement. P denotes phosphorylation. Yellow text boxes highlight key physiological processes that occur in the GC during signal transduction and stomatal opening. For further reference, see Lawson (2009); Daloso *et al.* (2016); Horrer *et al.* (2016); Santelia and Lawson (2016); Hiyama *et al.* (2017), and Inoue and Kinoshita (2017)

The red-light-driven opening response of stomata resembles the carbon assimilation response to increasing light intensity (Sharkey and Raschke, 1981) and is eliminated by inhibitors of photosynthetic electron transport, including 3-(3,4-dichlorophenyl)-1,1-dimethylurea (DCMU), indicating that it is photosynthesis dependent (e.g. Kuiper, 1964; Sharkey and Raschke, 1981; Tominaga *et al.*, 2001; Olsen and Junttila, 2002; Messinger *et al.*, 2006), suggesting that chlorophyll could be the receptor (Assmann and Shimazaki, 1999; Zeiger *et al.*, 2002). This red light response is considered the primary mechanism linking stomatal behaviour with mesophyll demands for CO_2_, although the exact location of the red light signal has not been fully elucidated. There are suggestions that it occurs in the chloroplast, either in the GCs themselves (Zeiger, 1983; Olsen *et al.*, 2002) or in the mesophyll, and that a signal is transferred from the mesophyll to the GCs (Lawson *et al.*, 2014). Several studies have suggested that GCs do not directly sense red light, and instead respond to the supply of CO_2_ in the mesophyll (see Fig. 2), coupling *A* and *g*_s_ via *C*_i_ (Mott, 1988; Roelfsema *et al.*, 2002), although other signalling mechanisms have been suggested (discussed below). Mesophyll consumption of CO_2_ driven by increasing photosynthetic irradiance reduces [CO_2_] in the intercellular air spaces to which stomata respond by opening; low light reduces this consumption and increases the concentration, closing stomata (Mott, 1988). Support for a *C*_i_-driven response, independent of a specific GC response, was provided by Roelfsema *et al.* (2001, 2002), who showed that a beam of blue light, but not red, induced changes in membrane potential in the GCs, altering K^+^ transport across the plasma membrane (Roelfsema *et al.*, 2001). Only when [CO_2_] around the GCs was altered under red light did these authors report that cells were hyperpolarized in CO_2_-free air, and switched to being depolarized when [CO_2_] was increased to 700 μmol mol^–1^, extruding K^+^ which resulted in stomatal closure (Roelfsema *et al.*, 2002). From these studies, it was concluded that GCs do not respond to red light directly but to the indirect changes in *C*_i_ (Roelfsema *et al.*, 2002). This is further supported by studies that have shown that stomatal opening to red light is mediated in part by a component of the low CO_2_ signalling network (HT1; HIGH LEAF TEMPERATURE1; Hashimoto *et al.*, 2006; Matrosova *et al.*, 2015), and that high [CO_2_] activates the release of Cl^–^ ions from GCs via S-type anion channels (such as SLAC1; Yamamoto *et al.*, 2016; Kusumi *et al.*, 2017; Zhang *et al.*, 2018), inducing stomatal closure (Fig. 2). Further studies showed that stomata of *Vicia faba* (Roelfsema *et al.*, 2006) and wheat (Karlsson, 1986*a*) did not respond to red light when treated with the carotenoid inhibitor norflurazon (which results in albino leaves that lack functional green chloroplasts). Similarly, no response was observed in the white area of variegated *Hedera helix* (Aphalo and Sanchez, 1986) and *Chlorophytum comosum* (that do contain photosynthetically active chloroplasts in the GC) when illuminated with red light (Roelfsema *et al.*, 2006). However, in both cases, stomata still responded to blue light, [CO_2_], and abscisic acid (ABA); therefore, it was concluded that photosynthetically active mesophyll is required for stomatal red light responses via changes in *C*_i_. However, many other studies have suggested that stomatal aperture responses to *C*_i_ are too small to account for the changes observed in *g*_s_ in response to light (Raschke, 1975; Sharkey and Raschke, 1981; Farquhar and Sharkey, 1982), and it has been reported that under red light, *g*_s_ increases even when *C*_i_ is held constant (Messinger *et al.*, 2006; Lawson *et al.*, 2008; Wang and Song, 2008), questioning *C*_i_ as the main driver of *A* and *g*_s_ coordination. Furthermore, studies on transgenic plants in which expression levels of several enzymes associated with electron transport or the Calvin cycle were manipulated leading to reduced photosynthetic rates demonstrated that stomata opened in response to light regardless of the higher *C*_i_ values observed (von Caemmerer *et al.*, 2004; Baroli *et al.*, 2008; Lawson *et al.*, 2008). The lack of coordination between *A* and *g*_s_ in these transgenic plants raised questions concerning the mechanism(s) that links these two processes, Other studies have suggested that an as yet unidentified signal originating in the mesophyll is potentially sensed by GCs activating a stomatal response. Wang and Song (2008) demonstrated that stomata on the abaxial leaf surface opened more widely when the leaf was irradiated from the adaxial rather than the abaxial side with *C*_i_ kept constant, supporting the idea of a direct mesophyll signal influencing stomatal behaviour. Lee and Bowling (1992, 1995) were the first to propose an aqueous metabolic signal, with potential candidates such as ribulose bisphosphate (RuBP), ATP, NADPH, malate, and sugar (Hedrich and Marten, 1993; Hedrich *et al.*, 1994; Zeiger and Zhu, 1998; Tominaga *et al.*, 2001; Lee *et al.*, 2008; Fujita *et al.*, 2013, 2019). However, further research has suggested a gaseous vapour phase ion (Mott *et al.*, 2008; Sibbernsen and Mott, 2010; Mott and Peak, 2013), sucrose metabolism (Lu *et al.*, 1997; Outlaw, 2003; Kang *et al.*, 2007), and even GC photosynthesis itself (Lawson *et al.*, 2003, 2014, 2018; Lawson, 2009).

### Guard cell osmoregulation in response to red light

The red light response (as with all stomatal responses) requires changes in osmotic potential in the GC, driven by the accumulation or loss of ions such as K^+^ and/or sugar accumulation (see reviews by Shimazaki *et al.* 2007; Lawson, 2009) to change water flux and therefore pore width (Fig. 2). Early research demonstrated that potassium accumulation is the result of red light activation of the plasma membrane proton pump (Serrano *et al.*, 1988; Olsen *et al.*, 2002), with ATP supplied by photophosphorylation in the GC chloroplasts (Shimazaki and Zeiger, 1985, Tominaga *et al.* 2001), although subsequent patch-clamp experiments could not replicate red light activation of the proton pump (Taylor and Assmann, 2001). Sugars as well as K^+^ have also been reported to accumulate in response to red-light-induced stomatal opening (Talbott and Zeiger, 1998; Olsen *et al.*, 2002), provided either by starch breakdown (Outlaw and Manchester, 1979), import from the mesophyll (e.g. Lu *et al.* 1995), or directly through GC photosynthesis (see review by Lawson, 2009). Although early studies on red-light-induced stomatal opening in epidermal peels reported high GC sucrose concentrations (Talbott and Zeiger, 1993), suggesting that the supply must be from GC photosynthetic carbon assimilation (Talbott and Zeiger, 1998), other studies have reported that GC photosynthesis is insufficient to produce sucrose required for osmoregulation (e.g. Outlaw, 1989; Reckmann *et al.*, 1990). This led to the hypothesis that apoplastic sucrose fixed in the mesophyll cells travels to the GCs via the transpiration stream (Lu *et al.*, 1995; Kang *et al.*, 2007), where it can be imported into the GCs via sucrose-mediated H^+^ symporter mechanism(s) (Daloso *et al.*, 2016) and act to open or close stomata or replace GC carbon stores (Lu *et al.*, 1997; Kelly *et al.*, 2013). Apoplastic sucrose accumulation at the GC has been proposed to initiate stomatal closure and provide a mechanism to coordinate *A* and *g*_s_ (Lu *et al.*, 1997; Ewert *et al.*, 2000; Outlaw and De Vlieghere-He, 2001). Outlaw and colleagues suggested that when mesophyll cells produce more sugar than can be loaded into the phloem, any excess will be carried to the GCs to reduce stomatal aperture (see Outlaw, 2003). This is supported by the numerous studies that have demonstrated sugar import into GCs (Reddy and Das 1986; Ritte *et al.*, 1999; Stadtler *et al.*, 2003; Weise *et al.*, 2008; Bates *et al.*, 2012; Daloso *et al.*, 2015; Antunes *et al.*, 2017). Kelly *et al.* (2013) suggested that sucrose arriving at the GC is cleaved in the apoplasts to produce glucose and fructose that is then sensed by hexokinase, which signals stomatal closure response. Although this mechanism may explain the reported decrease in *A* and *g*_s_ often observed over longer time scales and toward the end of the day (Vialet-Chabrand *et al.*, 2017b; Matthews *et al.*, 2018), it cannot explain the short-term coordination of *A* and *g*_s_.

### Role of guard cell chloroplasts in the red light response

It has recently been demonstrated that Arabidopsis mutants with reduced GC chlorophyll content have reduced *g*_s_, implying that GC photosynthesis is crucial for energetics and stomatal movements (Fig. 2; Azoulay-Shemer *et al.*, 2015). Support for the involvement of GC electron transport in light-induced stomatal opening also comes from work on the ‘crumpled leaf’ mutants, which lack GC chloroplasts. These mutants exhibited reduced levels of GC ATP and stomatal aperture in response to white light (Wang *et al.*, 2014). Wang *et al.* (2014) also demonstrated that lower ATP levels were observed in epidermal peels incubated (for 2 h in light) in isolation from the mesophyll, compared with peels that had been collected from intact leaf material after the incubation period. These findings strongly suggest that both GCs and mesophyll cells provide ATP for stomatal opening. Therfore, as there is currently no evidence for ATP import into GCs, mesophyll cells are likely to indirectly supply ATP by providing sugars that are utilized by the mitochondria (Wang *et al.*, 2014). The ATP from GC electron transport provides additional energy to that produced by glycolysis and mitochondrial respiration (Vavasseur and Raghavendra, 2005; Daloso *et al.*, 2016), which can be directly used for proton pumping or other metabolic processes involved in osmoregulation for stomatal opening in response to red (and blue) light (see Daloso *et al.*, 2015, 2016; Santellia and Lawson 2016; Santellia and Lunn, 2017). Interestingly, a recent report has demonstrated that red-light-induced plasma membrane H^+^-ATPase phosphorylation correlated with stomatal opening (Yamauchi *et al.*, 2016; Ando and Kinoshita, 2018), a process previously thought to be blue light dependent and under the control of the photoreceptor protein kinases, phototropins (see below). Using knockout mutants of one of the major isoforms of plasma membrane H^+^-ATPase in GCs, *aha*1-9, Ando and Kinoshita (2018) revealed that red-light-dependent stomatal opening was delayed in whole leaves. An immunohistochemical technique to detect phosphorylation demonstrated that DCMU inhibited plasma membrane H^+^-ATPase phosphorylation and red-light-induced stomatal opening. However, the lack of this response in isolated epidermal peels further suggests that mesophyll photosynthesis is required for the red light response. Furthermore, the authors did not rule out that GC chloroplasts might have the potential to induce partial phosphorylation of the plasma membrane H^+^-ATPase, and that this could be the underlying cause for interspecific differences in red light sensitivity in GCs. Moreover, as electron transport in the GCs and mesophyll chloroplasts is essentially the same, this could be involved in the regulation and coordination of *A* and *g*_s_ responses (Sharkey and Raschke, 1981; Lawson *et al.*, 2002, 2014; Messinger *et al.*, 2006; Lawson, 2009). Interestingly, the redox state of the chloroplastic plastoquinone pool (Q_A_) has been put forward as a signal that coordinates red light stomatal responses with the mesophyll at a range of light intensities (Busch, 2014). This was experimentally tested using tobacco mutants with reduced expression levels of PSII subunit S (PsbS), which directly effects the redox state of Q_A_ and results in a strong correlation with *g*_s_ when measured under a range of light intensities (Glowacka *et al.*, 2018). As tobacco generally lacks the GC-specific blue light response (see below), this enabled a direct relationship, driven by the red or photosynthetic light response, between Q_A_ and *g*_s_ to be evaluated.

## Stomatal blue light response

Blue-light-induced stomatal opening has been demonstrated in isolated epidermal peels and GC protoplasts (Zeiger and Helper, 1977); therefore, all the components required for this response are located in the GCs themselves (Kinoshita and Shimazaki, 2002; Ueno *et al.*, 2005; Hayashi *et al.*, 2011) and, unlike the red light response, does not require the involvement of a mesophyll signal (Ando and Kinoshita, 2018). Blue-light-induced stomatal opening is saturated at a low (~5–10 µmol m^–2^ s^–1^) fluence rate, too low to drive photosynthetic carbon gain, and is 20 times more effective at opening stomata than red light (Hsiao *et al.*, 1973; Karlsson, 1986*b*; Sharkey and Ogawa, 1987; Briggs, 2005). It has been proposed that the stomatal blue light response is important for morning pore opening to facilitate photosynthetic carbon gain early in the diel period, when the irradiance spectrum is enriched in blue wavelengths (Fig. 1; Zeiger, 1984). Additionally, this response could be important in rapid stomatal responses to sun flecks (Iino *et al.*, 1985) to maximize opportunistic periods of photosynthesis (Pearcy, 2007), particularly in understorey environments (Chazdon, 1988; Chazdon and Pearcy, 1991).

**Fig. 3.**
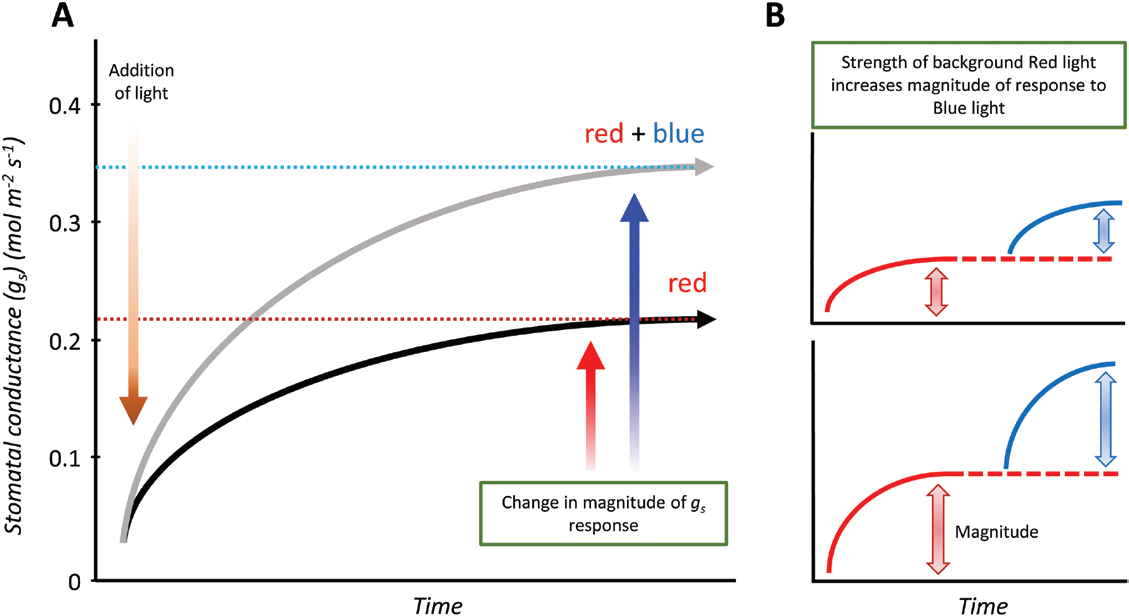
Schematic diagram of the impact of blue light on the magnitude of stomatal conductance (*g*_s_) compared with monochromatic red light. Highlighted is the impact of blue light on overall *g*_s_ (A); and the influence of different intensities of background red light on the magnitude of *g*_s_ response to blue light (B). Blue and red arrows indicate differences in *g*_s_ in response to blue and red light illumination.

Although the stomatal blue light response has been reported as not requiring photosynthesis, it has been demonstrated that two kinases found in the GC blue light signalling pathway—CBC1 and CBC2 (CONVERGENCE OF BLUE LIGHT AND CO_2_)—are actually involved in linking blue light responses (via phototropins) to low CO_2_ concentrations in the GC (Hiyama *et al.*, 2017; see Fig. 2). This therefore suggests that photosynthesis is indirectly involved, and in fact it has been shown previously that the sensitivity and magnitude of the stomatal response to blue light depend on the intensity of background red light (Karlsson, 1986*a*). In their review on light regulation of stomatal movement, Shimazaki *et al.* (2007) reported an increased *g*_s_ rate when blue light was applied to a background of red light compared with red alone, and virtually no response to weak blue light was observed when red light was absent, although this response could be species specific. Figure 3 highlights the impact of blue light on the magnitude of *g*_s_ compared with red light alone, demonstrating the independent and synergistic behaviour of the two distinct signals.

### Blue light signalling pathway

The stomatal blue light response is mediated by blue light photoreceptor protein kinases, known as phototropins (phot1 and phot2; Kinoshita and Shimazaki, 2001; Doi *et al.*, 2004; Christie *et al.*, 2007). Under blue light, phototropins within the GCs are activated via autophosphorylation and initiate a signalling cascade that eventually results in stomatal opening (see Fig. 2; Kinoshita and Shimazaki, 2001; Christie *et al.*, 2007; Shimazaki *et al.*, 2007; Inoue and Kinoshita, 2017). The protein kinase BLUE LIGHT SIGNALLING 1 (BLUS1) is directly phosphorylated by the activated phototropins (Takemiya *et al.*, 2013), and has been shown to indirectly transmit a signal to a type 1 protein phosphatase (PP1) and regulatory subunit PRSL1 (Takemiya *et al.*, 2006, 2013; Takemiya and Shimazaki, 2016). This blue-light-driven BLUS1 signal activates plasma membrane H^+^-ATPase in GCs via phosphorylation of a penultimate C-terminal residue (Thr) and through subsequent binding of a 14-3-3 protein (Shimazaki *et al.*, 2007; Hayashi *et al.*, 2011). Further recent research on the stomatal blue light pathway has identified that a Raf-like (receptor kinase involved in cell cycle regulation) protein kinase, BLUE LIGHT-DEPENDENT H^+^-ATPase PHOSPHORYLATION (BHP), binds to BLUS1 and forms a signalling complex with phototropins to mediate phosphorylation of plasma membrane H^+^-ATPase (Hayashi *et al.*, 2017). However, these authors suggest that BHP does not directly phosphorylate the penultimate Thr of membrane H^+^-ATPase, and another as yet unidentified signalling kinase may exist that directly controls phosphorylation of H^+^-ATPase, and stomatal opening in the blue light signalling cascade (Hayashi *et al.*, 2017; Inoue and Kinoshita, 2017). Blue-light-activated plasma membrane H^+^-ATPase initiates hyperpolarization of the membrane and drives H^+^ transport out of the GC (Shimazaki *et al.*, 2007). This hyperpolarization further activates inward-rectifying K^+^ channels, resulting in the influx and accumulation of K^+^ in the cytosol (Lebaudy *et al.*, 2008; Inoue and Kinoshita, 2017). Transport and accumulation of K^+^ and the counter-ions Cl^–^ and malate^2–^ into the vacuole occurs via K^+^/H^+^ exchangers (NHX1 and NHX2), chloride channel c (CLCc), and chloride channel malate transporters (ALMT9) (Jossier *et al.*, 2010; De Angeli *et al.*, 2013; Andrés *et al.*, 2014), which decrease GC water potential, increasing water uptake and turgor pressure, and ultimately leads to stomatal opening (Fig. 2; Inoue *et al.*, 2010; Eisenach and De Angeli, 2017; Inoue and Kinoshita, 2017; Jezek and Blatt, 2017). It has been suggested that among the phototropin-mediated responses, BLUS1 defines signalling specificity of stomatal opening and, as BLUS1 expression is only found in the GC and not in mesophyll cells, is not involved in other important phototropin-mediated responses (including phototropism and chloroplast movement) (Takemiya *et al.*, 2013; Inoue and Kinoshita, 2017), and therefore could be an unexploited target for manipulating stomatal behaviour.

In addition, the phototropin-dependent blue light signalling cascade and activation of plasma membrane H^+^-ATPase has been reported to be involved in carbon metabolism in GCs. A study by Horrer *et al.* (2016) revealed a novel pathway of starch degradation involving synergistic activities of β-amylase 1 (BAM1) and α-amylase 3 (AMY3) in GCs. This is in contrast to the mesophyll starch metabolism in which BAM3 is the major isoform and BAM1 has limited involvement (see Horrer *et al.*, 2016). Using the *phot1*/*phot2* double mutant and the BLUS1 mutant, these authors showed that the blue light signalling pathway was required for starch breakdown, in order to produce maltose which is subsequently turned into malate (which acts as a counter-ion for K^+^ uptake) through glycolysis and the activity of phosphoenolpyruvate carboxylase (PEPc; Horrer *et al.*, 2016). This is is agreement with other studies that have suggested a role for malate and PEPc in GC osmoregulation (Daloso *et al.*, 2016; Santelia and Lunn, 2017). This novel starch degradation pathway proposed by Horrer *et al.* (2016) highlighted that BAM1 and AMY3 are redox regulated (unlike the starch degradation enzymes associated with mesophyll activity), and suggested that GC electron transport could provide the reduced environment that would support BAM1 and AMY3 activation as well as providing a further connection between increasing light and stomatal opening, and a possible link between red and blue light responses (see below).

### Energetic and ATP supply for the guard cell blue light response

The fact that the blue light response has been observed in isolated tissues (Kinoshita and Shimazaki, 2002; Ueno *et al.*, 2005; Hayashi *et al.*, 2011) and occurs at low fluence rates has led to the suggestion that the ATP required for the proton pumps is most probably provided by GC mitochondria (Shimazaki *et al.*, 2007). This is supported by the lower number of chloroplasts (although this is species specific; Lawson *et al.*, 2003) and high concentration of mitochondria reported for GCs (Parvathi and Raghavendra, 1995; Willmer and Fricker, 1996) along with high rates of respiration (Allaway and Setterfield, 1972; Shimazaki *et al.*, 1983). Furthermore, when respiration was repressed with the inhibitors oligomycin and KCN or low [O_2_], ATP levels were greatly reduced in GCs (Shimazaki *et al.*, 1983; Gautier *et al.*, 1991), blue-light-dependent proton pumping was reduced (Mawson, 1993), and stomatal opening was inhibited (Schwartz and Zeiger, 1984). These findings demonstrate that the signalling pathways and at least some of the energetics for osmoregulation lie within the GCs themselves. In addition, photosynthetic electron transport within GC chloroplasts has been proposed to directly provide ATP for blue-light-induced H^+^ transport via plasma membrane H^+^-ATPase (Suetsugu *et al.*, 2014). In their experiment, Suetsugu *et al.* (2014) showed that red light enhanced blue-light-dependent H^+^ pumping in protoplasts, and that this was eliminated by DCMU, and in intact leaves DCMU inhibited both red and blue light stomatal opening. From this work, they concluded that ATP and/or reducing equivalents from GC electron transport is involved in fuelling blue-light-dependent stomatal opening.

## Impact of green light on stomatal behaviour and photosynthesis

Although the red and blue regions of the spectrum are considered the main drivers of photosynthesis and stomatal behaviour in higher plants, it is important to consider the influence of other spectral qualities of light, including green light. Green light (~500–560 nm) has been reported to inhibit blue-light-induced stomatal opening across a number of plant species (Talbott *et al.*, 2002), but seems to depend almost entirely on the light environment experienced by the plant during growth (Wang *et al.*, 2011; Aasamaa and Aphalo, 2016). Stomatal responses to green light were observed in plants grown under conditions reproducing an understorey environment (Aasamaa and Aphalo, 2016), and the magnitude of green-light-driven stomatal responses decreased over the course of the day (Talbott *et al.*, 2006). Although the receptors and exact mechanism of stomatal response to green light have not been identified, green light is known to deactivate the blue light cryptochrome photoreceptors via removal of the signal that suppresses ABA production in GCs, promoting a decrease in stomatal aperture (Bouly *et al.*, 2007). Green light has also been shown to contribute to photosynthesis, often at a more efficient rate than red or blue light due to non-photosynthetic absorption of blue light by carotenoids (McCree, 1972), and particularly in strong white light (Terashima *et al.*, 2009), and therefore can impact photosynthetic-dependent stomatal opening (Lanoue *et al.*, 2018). Moreover, Wang *et al* (2011) observed a green-light-driven stomatal response in sunflower leaves, which, similarly to the stomatal response to red light, was photosynthesis dependent as it was partly eliminated when DCMU was applied (Wang *et al.*, 2011). This implicates the potential existence of a green light receptor, with cryptochromes suggested as being involved in this DCMU-independent fraction (Lawson *et al.*, 2018), and that any further signal transduction is photosynthetically dependent, although further work would be required to elucidate a green light photoreceptor. It has been suggested that a possible role of the green light reversal effect on blue-light-driven stomatal opening could be the prevention of excessive leaf water loss through stomata under (green light-rich) vegetational shade, where, within a crop or other terrestrial plant canopy, photosynthetic potential is greatly reduced (Talbott *et al.*, 2006; Aasamaa and Aphalo, 2016).

## Species specificity to blue light

Interestingly stomatal responses to blue light are not universal, with fern species of *Polypodiopsida* (Doi *et al.*, 2015) and *Adiantum capillus-veneris* (Doi *et al.*, 2006), along with several species from the family Solanaceae, exhibiting a lack of stomatal response to blue light. The facultative CAM plant *Mesembryanthemum crystallinum* loses its stomatal blue light response when the plant shifts from C_3_ metabolism to CAM (Tallman *et al.*, 1997). In some species, including the gymnosperm *Cycas revoluta* and the ferns *Equiestum hyemale* and *Psilotum nudum*, blue light is essential for stomata to open (Doi *et al.*, 2015), suggesting that these differences may be due to various evolutionary pressures, whilst the loss of a stomatal blue light response in *Polypodiopsida* may be the result of adaption to understorey canopy environments (Doi *et al.*, 2015). Moreover, it should be kept in mind that growth conditions, such as drought and high temperatures that may alter the water status of the plant, may impact stomatal sensitivity to blue and red light (Wang *et al.*, 2011; Aasamaa and Aphalo, 2016; Lanoue *et al.*, 2018). This is because plants will balance the need to maintain leaf turgor and/or maximize carbon gain and evaporative cooling (Lawson and Blatt, 2014), and finding the balance between these factors is ultimately dependent on species specificity to the growth environment (Lawson and Vialet-Chabrand, 2019). Although several species have been reported to respond to blue light (e.g. *Arabidopsis thaliana* and *Vicia faba*; Table 1), those that do not respond or exhibit a diminished or slow response have generally not been reported (e.g. *Nicotiana tabacum*; Loreto *et al.*, 2009). This is further complicated by the different protocols used to assess red and blue light responses, making comparison almost impossible, with different red and blue light intensities, ratios, and even durations being applied. Furthermore, the photosynthesis dependence of stomatal response to blue light is species specific (see Wang *et al.*, 2011; Dumont *et al.*, 2013; Suetsugu *et al.*, 2014), with some species known to respond to blue light even in the dark (with no red light background) (Dumont *et al.* 2013), whilst in others stomatal blue light is only apparent on a background of red light. This has significance, as it is presumed that the response of stomata to blue light does not necessarily require photosynthesis, and that different species may use different sources of energy for blue-light-induced stomatal opening (Daloso *et al.*, 2016; McLachlan *et al.*, 2016; Santelia and Lunn, 2017). Given the evidence that indicates that species specificity of stomatal response to blue light exists, there is a need for standardization of the protocols to be able to accurately compare the biological importance of the stomatal blue light response between and within species.

**Table 1.** Species-specific response to the addition of blue light

Species	Response	Publications	Measurement	Intensity of RL (μ mol m^–2^ s^–1^)	Intensity of BL (μ mol m^–2^ s^–1^)	Duration of BL (min)	Approximate increase (%)
Model plants							
*Arabidopsis thaliana*	Yes	Talbott *et al.* (2002)	*a*	0	5	90	–
		Takemiya *et al.* (2013)	*g*_sw_	80/600	5	20	75
		Suetsugu *et al.* (2014)	*g*_sw_	60/240/600	5	10	50
		Doi *et al.* (2015)	*g*_sw_	600	5	5	30
*Nicotiana glauca*	Yes	Talbott *et al.* (2002)	*a*	0	5	90	–
*Nicotiana tabacum*	Yes	Talbott *et al.* (2002)	*a*	0	5	90	–
	No	Loreto *et al.* (2009)	*g*_sw_	210	90	30	0
Crops							
*Triticum aestivum*	Yes	Karlsson *et al.* (1983)	*E*	0	20/50/100	120	–
		Karlsson (1986*a*)	*g*_sw_	460	25	2	30
*Oryza sativa*	Yes	Shimazaki *et al.* (2007)	*g*_sw_	600	5	20	33
*Helianthus annuus*	Yes	Wang *et al.* (2011)	*g*_sw_	0	250	30	100
*Hordeum vulgare*	Yes	Talbott *et al.* (2002)	*a*	0	5	90	–
*Vicia faba*	Yes	Lurie (1978)	*a*	0	20	150	33
		Ogawa (1981)	*E*	–	–	30	50
		Assmann *et al.* (1985)	*g*_sw_	525	260	0.83	19
		Gorton *et al.* (1993)	*g*_sw_	0	150	90	50
		Frechilla et al. (2000, 2004)	*a*	120	10	90	50
		Talbott *et al.* (2002)	*a*	0	5	90	–
		Takemiya *et al.* (2006)	*a*	150	10	150	50
*Pisum sativum*	Yes	Talbott *et al.* (2002)	*a*	0	5	90	–
*Lactuca sativa*	Yes	Clavijo-Herrera *et al.* (2018)	*g*_sw_	270	19	–	50
*Allium cepa*		Ogawa (1981)	*E*	–	–	30	50
		Talbott *et al.* (2002)	*a*	0	5	90	–
Trees							
*Nothofagus alpina* (Popp. and Endl.) Oerst	Yes	Aasama and Aphalo (2016)	*g*_sw_	250	15	6	50
*Betula pendula* Roth	No	Aasama and Aphalo (2016)	*g*_sw_	250	15	6	0
*Populus deltoides×Populus nigra*	Yes	Dumont *et al.* (2013)	*g*_sw_	0	30	40	165
*Platanus orientalis*	No	Loreto *et al.* (2009)	*g*_sw_	210	90	30	0
*Ginkgo biloba*	Yes	Doi *et al.* (2015)	*g*_sw_	600	5	60	30
Ferns							
*Dicranopteris linearis*	No	Doi *et al.* (2015)	*g*_sw_	600	5	60	0
*Angiopteris lygodiifolia*	Yes	Doi *et al.* (2015)	*g*_sw_	600	5	60	15
*Botrychium ternatum*	Yes	Doi *et al.* (2015)	*g*_sw_	600	5	60	43
*Equisetum hyemale*	Yes	Doi *et al.* (2015)	*g*_sw_	600	5	60	300
*Psilotum nudum*	Yes	Doi *et al.* (2015)	*g*_sw_	600	5	60	1150
*Lepisorus thunbergianus*	No	Doi *et al.* (2015)	*g*_sw_	600	5	60	0
*Thelypteris acuminata*	No	Doi *et al.* (2015)	*g*_sw_	600	5	60	0
*Osmunda japonica*	No	Doi *et al.* (2015)	*g*_sw_	600	5	60	0
*Alsophila mertensiana*	No	Doi *et al.* (2015)	*g*_sw_	600	5	60	0
Lycophytes							
*Selaginella moellendorffii*	Yes	Doi *et al.* (2015)	*g*_sw_	600	5	60	50
*Selaginella uncinata*	Yes	Doi *et al.* (2015)	*g*_sw_	600	5	60	80
Others							
*Commelina communis*	Yes	Iino *et al.* (1985)	*g*_sw_	500	25	90	54
		Assmann (1988)	*g*_sw_	263	100	15	36
		Assmann (1992)	*g*_sw_	0/700/1500	200	43	60
		Lascève *et al.* (1993)	*g*_sw_	105	65	1	115
		Talbott *et al.* (2002)	*a*	0	5	90	–
*Paphiopedilum harrisianum*	Yes	Zeiger *et al.* (1983)	*a*	0	10	120	300
		Assmann (1988)	*g*_sw_	263	100	15	33
*Mesembryanthemum crystallinum*	Yes	Mawson and Zaugg (1994)	*a*	0	300	110	43
		Tallman *et al.* (1997)	*a*	350	15	240	–
*Xanthium pennsylvanicum*	Yes	Mansfield and Meidner (1966)	*g*_sw_	–	–	240	700
*Tradescantia pallida*	Yes	Ballard *et al.* (2019)	*a*	50	50	–	–
*Musa acuminata* cv. *G*rand Nain AAA	Yes	Zait *et al.* (2017)	*g*_sw_	1080	120	120	40
*Festuca arundinacea*	Yes	Barillot *et al.* (2010)	*g*_sw_	277	60	–	100
*Cycas revoluta*	Yes	Doi *et al.* (2015)	*g*_sw_	600	5	60	3900
*Chamaecyparis obtusa*	Yes	Doi *et al.* (2015)	*g*_sw_	600	5	60	50
*Gnetum* spp.	Yes	Doi *et al.* (2015)	*g*_sw_	600	5	60	120
*Zamia furfuracea*	Yes	Doi *et al.* (2015)	*g*_sw_	600	5	60	100
*Phragmipedium longifolium*	Yes	Zeiger *et al*. (1985)	*g*_sw_	65	85	15	30
*Paphiopedilum insigne*	Yes	Zeiger *et al*. (1985)	*g*_sw_	68	82	15	20

The presence or absence of the response was reported for each species as well as the experimental protocol used. Measurements are reported as stomatal aperture (*a*), stomatal conductance (*g*_sw_), or transpiration (*E*).

## Impact of red and blue light on dynamic stomatal response

**Fig. 4.**
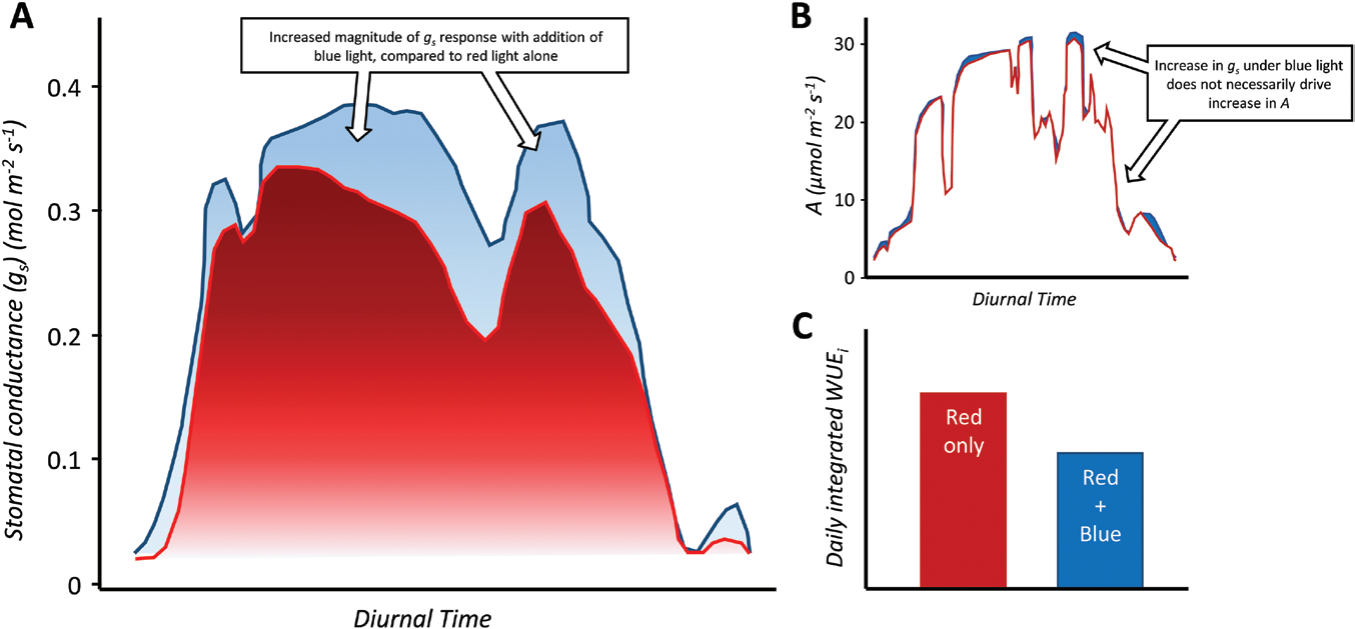
Influence of the addition of blue light on the magnitude of stomatal conductance (*g*_s_) over the diurnal period. Using *Triticum aestivum* (common wheat) as an example, highlighted is the impact of blue light compared with monochromatic red light on: the magnitude of *g*_s_ (A) and net photosynthetic rate (*A*) (B) over the diurnal period; and the consequential impact for daily water use efficiency (daily integrated WUE_i_) (C). As the percentage of blue light is higher early in the diel period, *g*_s_ response to blue light is enhanced. Evidence suggests that blue light maintains *g*_s_ levels throughout the diel period (A), although this does not necessarily increase carbon gain (B), and therefore greatly reduces daily water use (C)

**Fig. 5.**
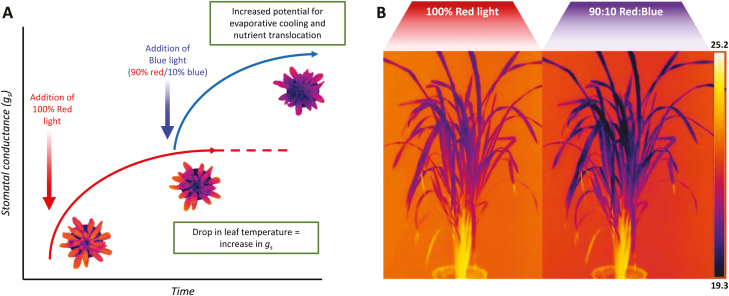
Theoretical representation of the impact of blue-light-driven changes on stomatal conductance (*g*_s_) on leaf temperature and evaporative cooling. Representative courses of *g*_s_ under a step increase in red light and a further addition of blue light (the total light intensity remains constant) (A). *Arabidopsis thaliana* plants subjected to these light conditions are shown, highlighting the change in leaf temperature driven by changes in *g*_s_ (A). Representative rice (*Oryza sativa*) plants exposed to 30 min of 100% red light and a 90:10 ratio of red to blue light, demonstrating the difference in leaf temperature (°C) and therefore *g*_s_ in a major crop variety (B).

Although some species do not exhibit a stomatal blue light response (Doi *et al.*, 2006), little is known about diversity in the magnitude and/or speed of these responses. This is especially important when considering the impact stomatal behaviour to blue light might have on carbon uptake and water use in major crop species (see Wang *et al.*, 2011; Dumont *et al.*, 2013; Suetsugu *et al.*, 2014). The fact that stomata in some species open to blue light even when photosynthesis is already saturated with red light (Shimazaki *et al.*, 2007) means that *g*_s_ may be higher than required to achieve maximum CO_2_ diffusion for photosynthesis, and therefore WUE is greatly reduced. Conversely, the impact of stomatal opening response to blue light on carbon uptake depends on the degree of diffusional limitation of *g*_s_ for photosynthesis. Figure 4 illustrates the influence of blue light on *g*_s_ over the diurnal period, and how this greatly affects WUE even when photosynthesis is saturated. From this, we can infer that reducing stomatal sensitivity to blue light may potentially be beneficial for optimizing crop resource use, whereby photosynthetic rates are maintained whilst using water more efficiently. However, this may only be beneficial under certain environmental conditions, as reduced *g*_s_ could lead to increased leaf temperature, which, depending on the species and environment, could be detrimental to photosynthetic rates (Matthews and Lawson, 2019) and overall plant productivity. Here we show, via thermal imaging, the impact of blue-light-dependent *g*_s_ response on leaf cooling, and how the addition of ~10% of blue light facilities greater leaf evaporative cooling even when light intensity is held constant (Fig. 5). Decreasing water loss during early stages of growth in crops such as wheat would facilitate greater water availability later in the season, that in turn would enable sustained photosynthetic rates through the grain-filling period when water is a major limiting factor, potentially increasing overall grain yield (Acreche and Slafer, 2009; Carmo-Silva *et al.*, 2017; Kaiser *et al.*, 2018; Lanoue *et al.*, 2018).

Generally, the mechanisms behind the speed of stomatal response refer to short-term responses (seconds to minutes), and are not necessarily sufficient to explain diurnal behaviour of *A* and *g*_s_. Even in constant light conditions, decreases in *g*_s_ are often seen towards the end of the day (Matthews *et al.*, 2018), and it has been suggested that mechanisms such as increases in the amount of sucrose from photosynthesis mediate this response, and that sucrose content and metabolism play a major role in the longer term coordination of *A* and *g*_s_ (Lawson *et al.*, 2014). This sugar accumulation at high photosynthetic rates associated with high photosynthetically active radiation (PAR) conveys long-term photosynthetic feedback on *g*_s_ over the course of the day (Lu *et al.*, 1995, 1997; Outlaw, 2003; Kang *et al.*, 2007; Kelly *et al.*, 2013), which may theoretically alter stomatal dynamic behaviour over the diurnal period (Matthews *et al.*, 2017). It should also be noted that toward the end of the day, some species exhibit a slower *g*_s_ response to changes in light intensity, with a slow closing response resulting in continued high *g*_s_, leading to substantial water loss and reduced WUE over the diurnal period (Blom-Zandstra *et al.*, 1995; Lawson and Blatt, 2014). It has already been reported that in wheat the magnitude or sensitivity of stomata to blue light is enhanced under a ‘strong’ red light background (Karlsson, 1986*a*), with this behaviour potentially being dose dependent where the intensity and even the duration of the background red light determines the extent to which stomata respond to a blue light signal (Ogawa *et al.*, 1978; Zeiger, 1984; Iino *et al.*, 1985; Karlsson, 1986*a*; Sharkey and Ogawa, 1987; Assmann, 1988). Furthermore, as the stomatal response to blue light is GC specific, it may be suggested that the speed of the *g*_s_ response is increased under blue light. It can therefore be proposed that altering the sensitivity and diurnal behaviour of the *g*_s_ blue light response, via either breeding techniques or genetic modification, could lead to a reduction in the limitation of *A* by *g*_s_, and the slow decrease in *A* and *g*_s_ through the day may be prevented. This paves the way for potential improvements in photosynthetic carbon assimilation over the diurnal period (Vialet-Chabrand *et al.*, 2017b; Matthews *et al.*, 2017, 2018), whilst positively influencing WUE and plant productivity. As most studies have been carried out under ‘ideal’ well-watered conditions, there is little information describing the influence of drought, water status, or temperature on the temporal response of *g*_s_ (Lawson and Blatt, 2014; Haworth *et al.*, 2018). As a consequence, it is currently unknown how manipulation of stomatal sensitivity to blue and red light may impact plant fitness and productivity. However, as the frequency and intensity of periods of drought are set to increase globally in the near future, water availability and its transport from roots to stomata will be a major limiting factor for crop and terrestrial ecosystems moving forward. As such, improving our understanding of the dynamic response of stomata to different spectra of light and how manipulation of the stomatal sensitivity will impact spatial and temporal stomatal responses (Matthews *et al.*, 2017; Vialet-Chabrand *et al.*, 2017a; Lawson and Vialet-Chabrand, 2019; Vialet-Chabrand and Lawson, 2019), remains an unexploited avenue in which to improve plant performance and crop productivity.

## Summary and future perspectives

Stomatal research over the past few decades has revealed a complicated network of osmoregulatory and signalling pathways in GCs (e.g. Lawson, 2009; Daloso *et al.*, 2016; Inoue and Kinoshita, 2017) that are species specific and influenced by the growth environment. Although significant progress has been made over the past few decades, understanding of stomatal responses to various environmental signals (including irradiance) and substantial advances in GC metabolism and osmoregulatory pathways, many gaps remain regarding the integration and hierarchy of these diverse processes and the extent to which each contributes to stomatal function. As we have illustrated throughout this review, there is extensive evidence for both mitochondrial and photosynthetic electron transport ATP supply for the H^+^-ATPase, ion channel activation, and stomatal opening in response to both blue and red illumination. However, the extent to which each energetic supply contributes to stomatal movements has yet to be fully elucidated. It is also apparent that manipulating one pathway may result in the up-regulation of an alternative pathway to compensate, increasing the difficulty and complexity for determining the involvement and extent of each. The role and participation of GC electron transport and photosynthetic processes remain continuing subjects of debate, despite the fact that chloroplasts are a key feature of most GCs. Furthermore, the extent to which mesophyll signals play a role and the origins and nature of these signals need clarification. Therefore, understanding the mechanisms and signal transduction pathways that operate in GCs and the influence of mesophyll photosynthesis on these processes (and subsequent stomatal responses) is essential if we are to fully exploit the relationship between *A* and *g*_s_ in order to improve gaseous fluxes, maximize CO_2_ uptake, and optimize water use in fluctuating environments given the predicted changes in climate (Matthews and Lawson, 2019).

We are all fully aware that global demand for food is growing, and, due to a growing world population, it has been estimated that a >50% increase in major crop yield is required by 2050 (Long *et al.*, 2015). This situation is further exacerbated by predicted increases in atmospheric temperature and heat wave frequency experienced by crop and terrestrial ecosystems (Perkins *et al.*, 2012). These variable changes in climate conditions are often linked to changes in precipitation patterns, water availability (Stéfanon *et al.*, 2014), and drought (Urban *et al.*, 2017), and are set to aggravate crop losses and increase the agricultural water supply requirements by an estimated 17% (Pennisi, 2008). When water is limiting, plants close stomata to avoid excessive water loss, even during periods of high light when mesophyll demands for CO_2_ are high, and long-term damage to photosynthetic machinery may be induced (Berry and Björkman, 1980). In crop and forest ecosystems this reduces transpiration but at a cost of reduced evaporative cooling (Ainsworth and Rogers, 2007; Bernacchi *et al.*, 2007; Lammertsma *et al.*, 2011; Hussain *et al.*, 2013; Tricker *et al.*, 2018), which greatly impacts biochemical and metabolic mechanisms of photosynthesis (Perdomo *et al.*, 2017); including but not limited to the activity of the temperature-sensitive enzyme Rubisco activase and ATP synthesis (Tezara *et al.*, 1999; Galmés *et al.*, 2007). Finding the ‘ideal’ balance between carbon gain, evaporative cooling, and maintenance of hydraulic status is crucial for maximizing crop performance and productivity, whether it be for field- or greenhouse-grown crops.

As mentioned above, blue light induces a GC-specific stomatal response that enables stomatal opening, increasing the magnitude of *g*_s_. Given the importance of the blue light response of *g*_s_ for evaporative cooling and carbon gain in major crop species (see above), manipulation of this blue-light-triggered response represents a novel and generally unexploited target as a strategy to increase carbon uptake and/or maintenance of optimal leaf temperature, and conversely for crop water use. An analogous approach has recently been used by Papanatsiou *et al.* (2019) who expressed the synthetic light-gated K^+^ channel BLINK1 specifically in the GCs to enhance solute fluxes, and produced plants with stomata that opened and closed more rapidly, resulting in greater WUE and biomass.

Understanding the mechanisms behind the blue- and red-light-driven responses of stomata would potentially enable greater control of the synchronicity between *A* and *g*_s_ under dynamic light conditions, and therefore optimize the relationship between water use and carbon gain (Lawson and Blatt, 2014). Enabling a blue light *g*_s_ response in species in which it is absent increases the potential for cultivating crop ideotypes for specific climate conditions. In fact, it has already been demonstrated that blue light, even at low intensities, reduced stomatal oscillations often seen under drought conditions (Zait *et al.*, 2017; Ballard *et al.*, 2019). These oscillations, the cyclic opening and closing of stomata, are presumed to initiate from hydraulic mismatch between water supply and transpiration rate, and therefore it is suggested that a blue-light-driven *g*_s_ response helps recover this synchronicity and improve plant performance under drought (Zait *et al.*, 2017). On the other hand, reducing stomatal sensitivity to blue light could provide a route to producing plants with reduced levels of *g*_s_, potentially enhancing water saving at crucial stages during plant development (e.g. grain filling). However, this could be to the detriment of CO_2_ diffusion and leaf cooling, but could provide ideotypes for specific growth environments.

Given the increase in alternative growth spaces (e.g. vertical farming) for ‘indoor’ crops, a new generation of smart LED lighting allowing for more precise control of light quality and quantity has recently become available. Several recent studies have demonstrated the importance of blue light for vegetable crop growers, as a way of improving WUE and even overall yield (Clavijo-Herrera *et al.*, 2018; Kaiser *et al.*, 2018; Lanoue *et al.*, 2018; Pennisi *et al.*, 2019). For example, a recent study demonstrated that different ratios of red and blue light optimized growth, yield, and WUE in basil (Pennisi *et al.*, 2019). This is interesting, as the optimal ratios for these targets were shown to be different from the ratios of light spectra observed in nature, whether it is direct or diffuse light (Urban *et al.*, 2007). This highlights the potential to engineer the tapestry of plant pigments to utilize more of the sunlight’s spectrum, to maximize light absorption, and to overcome light saturation of the downstream photosynthetic processes (Long *et al.*, 2015; Song *et al.*, 2017), as it is already known that more than half of the energy in the solar spectrum is not utilized by the plant (Zhu *et al.*, 2008).

In this review, we have focused on stomatal response to different light qualities. Emphasis is placed on the response of *g*_s_ to blue and red spectra of light, and how plants use these independent light responses to enforce GC movement to maximize plant performance. With recent research highlighting the importance of the rapidity of *g*_s_ responses to light for plant water status and photosynthetic carbon gain, we emphasize the need to re-assess the role of stomatal behaviour under blue and red light between and within species, as a means to understand the importance of stomata in crop performance. Additionally, with increases in global temperature and water demand for agricultural practices predicted to intensify crop losses in the future, we outline the potential of manipulating stomatal response to light quality to maximize drought tolerance, water-saving strategies, and yield.
